# Draft Genome Sequence of a Phthalate Ester-Degrading Bacterium, *Rhizobium* sp. LMB-1, Isolated from Cultured Soil

**DOI:** 10.1128/genomeA.00392-15

**Published:** 2015-05-07

**Authors:** Wen-Juan Tang, Ying Zhou, Bang-Ce Ye

**Affiliations:** Lab of Biosystems and Microanalysis, State Key Laboratory of Bioreactor Engineering, East China University of Science and Technology, Shanghai, China

## Abstract

*Rhizobium* sp. LMB-1, newly isolated from greenhouse soil, can effectively degrade phthalate. Here, we present a 5.2-Mb assembly of this *Rhizobium* sp. genome for the first time. It may provide abundant molecular information for the transformation of phthalates.

## GENOME ANNOUNCEMENT

*Rhizobium* is a genus of Gram-negative soil bacteria that fix nitrogen, a natural fertilizer for plants (1–3). However, there is no research concerning the biodegrading ability of phthalate esters (PAEs). In this study, *Rhizobium* sp. LMB-1 was isolated from greenhouse soil, which was recognized as utilizing PAEs as the sole carbon source, and optimal conditions of biodegradation were determined. The results demonstrated that the bacterium LMB-1 is a novel candidate in the application of PAE bioremediation in agricultural soil. The capacity of the novel *Rhizobium* isolate LMB-1 degraded high concentrations of phthalate (DMP), diethyl phthalate (DEP), dibutyl phthalates (DBP), and di(2-ethylhexyl) phthalate (DEHP). The optimal degradation conditions of DEHP were pH 6 and 37°C.

In our laboratory, significant cell growth was observed in a tryptic soy broth medium. Analysis of 16S rRNA gene sequences from single colonies allowed the assignment of the isolate to the genus *Rhizobium*, with the highest sequence identity (99%) observed with the 16S rDNA gene of *Rhizobium pusense*. The genus *Rhizobium* consists of Gram-negative, strictly aerobic, chemo-heterotrophic, rod-shaped, and yellow-pigmented bacteria (4). It is one of three closely related genera (*Rhizobium pusense* strain NRCPB10, *Agrobacterium fabrum* strain C58, *Rhizobium nepotum* strain 39/7) into which the previous, more comprehensive genus *Rhizobium* was subdivided on the basis of 16S rDNA gene sequences and biochemical features.

DNA extracted from a single colony of *Rhizobium* sp. LMB-1 was subjected to whole-genome sequencing, and 300-bp paired-end libraries were generated. Sequencing was done using Illumina’s TurSeq DNA sample prep kits according to their protocols and sequenced on Illumina MiSeq machines by the Majorbio Company. The insert sizes averaged 300 nucleotides, and 1,788,155 × 2 reads were obtained, comprising approximately 162× coverage of the genome. Read assembly was performed *de novo* using SOAPdenovo version 2.04 (http://soap.genomics.org.cn) (5). Several *k*-mers were run, and the best resulting assembly was chosen based on assembly contiguity statistics, the placement of a subset of high-quality read pairs in the assembly with correct spacing, and orientation.

The draft genome sequence is 5.22 Mb in length (59.9% G+C content) and consists of 73 contigs. Scaffolding produced 66 supercontigs, of which the largest one (0.97 Mb) represented 18% of the total assembly length. Genes were predicted by using Glimmer version 3.02, and 4,959 protein coding genes, 39 tRNA genes, and 1 rRNA operon were identified.

### Nucleotide sequence accession number.

The genome sequences have been deposited in NCBI GenBank under the accession number JZUD00000000.
